# On the Feasibility of Wireless Multimedia Sensor Networks over IEEE 802.15.5 Mesh Topologies

**DOI:** 10.3390/s16050643

**Published:** 2016-05-05

**Authors:** Antonio-Javier Garcia-Sanchez, Fernando Losilla, David Rodenas-Herraiz, Felipe Cruz-Martinez, Felipe Garcia-Sanchez

**Affiliations:** 1 Department of Information and Communication Technologies, Universidad Politécnica de Cartagena (UPCT), Campus Muralla del Mar, E-30202 Cartagena, Spain; fernando.losilla@upct.es (F.L.); felipecruz91@hotmail.es (F.C.-M.); felipe.garcia@upct.es (F.G.-S.); 2 Computer Laboratory, University of Cambridge, William Gates Building, 15 JJ Thompson Avenue, Cambridge CB3 0FD, UK; dr424@cl.cam.ac.uk

**Keywords:** WMSN, IEEE 802.15.5, mesh topology, multimedia, WSN

## Abstract

Wireless Multimedia Sensor Networks (WMSNs) are a special type of Wireless Sensor Network (WSN) where large amounts of multimedia data are transmitted over networks composed of low power devices. Hierarchical routing protocols typically used in WSNs for multi-path communication tend to overload nodes located within radio communication range of the data collection unit or data sink. The battery life of these nodes is therefore reduced considerably, requiring frequent battery replacement work to extend the operational life of the WSN system. In a wireless sensor network with mesh topology, any node may act as a forwarder node, thereby enabling multiple routing paths toward any other node or collection unit. In addition, mesh topologies have proven advantages, such as data transmission reliability, network robustness against node failures, and potential reduction in energy consumption. This work studies the feasibility of implementing WMSNs in mesh topologies and their limitations by means of exhaustive computer simulation experiments. To this end, a module developed for the Synchronous Energy Saving (SES) mode of the IEEE 802.15.5 mesh standard has been integrated with multimedia tools to thoroughly test video sequences encoded using H.264 in mesh networks.

## 1. Introduction

Wireless Sensor Networks (WSNs) [1] consist of a large number of low-cost and energy-constrained devices. These devices transmit data acquired by a number of in-built sensors to one or several data collection units, named data sinks, which are in charge of sending data to remote database servers for further data processing and storage. Wireless Multimedia Sensor Networks (WMSNs) [2] are regarded as a particular type of WSN where nodes are interfaced with sensors for visual and sound data acquisition, such as Complementary Metal Oxide Semiconductor (CMOS) cameras and microphones. This technology has drawn the attention of the WSN research community, encouraged by the challenge of providing support applications such as video surveillance, environmental monitoring, target tracking and traffic management, where multimedia services are required.

Compared with low-data-sampling, low-data-transmission rate WSN applications, multimedia applications impose stringent requirements in terms of high sampling rate, data transmission reliability, data latency and data throughput. Reliable transmission of multimedia data in WMSNs is challenging due to the inherent technology limitations, such as interference between devices or limited data transmission bandwidth. Multimedia communications also require a high utilization of the transmission channel, increasing communication activity within the network and considerably decreasing the operational lifetime of battery-powered nodes. This clearly contrasts with the traditional WSN/WMSN requirements imposed by, on the one hand, the use of short-range communications over low-bandwidth links (maximum bitrate of 250 kbps at 2.4 GHz) and, on the other hand, the energy constraints of sensor devices.

Traditional routing in WSNs is also a problem in WMSNs. Many state-of-the-art multi-hop proposals for WSNs consider tree-based topological configurations where only a few number of nodes are likely to forward all the data traffic. Because of the intensive use of these nodes, the network may not fulfil the Quality of Service (QoS) required by the multimedia application and, more importantly, it will lead to the overuse and depletion of the batteries of nodes in the path. In addition, traditional routing mechanisms usually only deal with the transmission to a sink node and not to any arbitrary node of the network, which hampers the development of collaborative applications or the use of multiple sinks.

Mesh topologies, in addition to enabling multiple routing paths between two nodes outside of radio communication range with each other, have proven advantages against other topological configurations, such as tree-based or cluster-based topologies, which include data transmission reliability, network robustness against node failures, and potential reduction in energy consumption [3]. Nodes arranged in a mesh topology are generally easier to deploy and potentially easier to maintain as they are able to reconfigure themselves in order to adapt to the conditions brought by the deployment environment. Among the different mesh solutions available for low-power wireless networks, the low-rate part of the IEEE 802.15.5 (hereafter IEEE 802.15.5) [4] offers suitable functionalities for enabling multimedia applications compared with other state-of-the-art network/MAC layer standards. This standard assures that any node is able to communicate with any other node of the network through different routes, enhancing data transmission reliability and robustness against node failures. It also emphasizes simplicity, adding, for low-rate WPAN, a thin mesh sublayer on top of the IEEE 802.15.4 MAC sublayer, the most widely adopted point-to-point communication standard for low-rate wireless personal area networks (LR-WPANs). This results in an easy migration of applications using the 802.15.4 standard and in a greater scalability, enabling large-scale deployments. In addition, the standard provides two radio duty-cycle mechanisms, named Asynchronous Energy Saving (ASES) and Synchronous Energy Saving (SES). These mechanisms are intended to make efficient use of the available battery energy for, respectively, asynchronous and synchronous communications, thereby prolonging the battery life. For all the aforementioned reasons, IEEE 802.15.5 can be, a priori, a suitable and representative WMSN solution for the transmission of video in mesh topologies.

However, according to our knowledge, there are very few studies in the scientific literature combining mesh topologies with multimedia applications for low power devices. Under these circumstances, the purpose of this paper is to contribute to the development of multimedia applications in the WMSN arena, fulfilling the WSN/WMSN specific requirements, assuring the QoS of the multimedia service and achieving a reasonable performance of the network in terms of, for instance, lifetime and data transmission reliability. To achieve these goals, we have designed a novel tool, available for download, that evaluates, by means of computer simulation, the performance of video transmissions using the SES energy-saving mechanism of IEEE 802.15.5. For the evaluation of the standard, Quarter Common Intermediate Format (QCIF) video sequences are encoded using the H.264/AVC compression standard and transmitted through WMSN mesh networks of different sizes. The main results will be discussed, highlighting the most important metrics such as latency, jitter, time between packets, message delivery ratio, power consumption, lifetime and Peak Signal-to-Noise Ratio (PSNR). The rest of this paper is organized as follows: Section 2 introduces related work in the field of WMSNs. Section 3 provides the necessary technological background about video encoding with H.264 as well as WMSN operation based on the IEEE 802.15.5 standard. Section 4 describes the simulation tool and simulation environment. Section 5 shows and analyzes simulation results, which are further discussed in Section 6. Section 7 concludes this work.

## 2. Related Work

In spite of the proven advantages arising from the use of the IEEE 802.15.5 standard, to the knowledge of the authors, there is no other work that makes use of this technology in WMSNs. There are however a number of proposals that use mesh communication for transmitting multimedia flows [5]. However, they do not consider Wireless Sensor Networks but rather focus on less energy-constrained standards such as 802.11. In this regard, the purpose of this work is twofold: first, to carry out a first evaluation of the performance of a mesh standard, the IEEE 802.15.5, for video transmission; second, to provide simulation tools to help evaluate further improvements for multimedia transmission made to the original standard.

The performance of WSNs and WMSNs should be assessed using real WSN/WMSN hardware before actual deployment, for example, using a test bed in the laboratory. However, it is best practice to make sure that no software/hardware errors are introduced before test-bed experiments are carried out. Finding and correcting these errors is usually a tedious and time-consuming task, and simulation is a powerful tool that helps do this in a much faster and easier way than dealing directly with hardware. In this regard, Pham [6] studied the communication performance of the most popular WSN commercial platforms to date in data-intensive applications. Results showed significant delays limiting the maximum data throughput at both the sender and the receiver. The best results were obtained using MicaZ devices [7]. Pham also showed that more effort should be made on the development of new hardware platforms, but also that more realistic simulation models are needed to consider the constraints of these commercial devices. Farooq *et al.* [8] studied the feasibility of performing the evaluation of new multimedia proposals in large WSN testbeds available for research usage. They concluded that, despite the existence of powerful hardware platforms for multimedia applications, there has been little work yet to integrate them into testbeds. Consequently, simulations are the most cost-effective solution for evaluating WMSN proposals.

Some researches focused on comparing the performance of data-intensive WMSN applications using different MAC and network protocols for diverse layouts. Ammar *et al.* [9] compared the performance obtained in WMSN by using two well-known WSN MAC protocols, S-MAC and 802.15.4, and two routing protocols, Ad hoc On-Demand Distance Vector (AODV) routing and its extension Ad hoc On-demand Multipath Distance Vector (AOMDV) routing. They remarked on the importance of optimizing the network layer protocols. In addition, their simulation results showed a better performance of the IEEE 802.15.4 standard in terms of network lifetime, which is supported by the large number of WMSN proposals based on this standard. Andrea *et al.* [10] studied this standard in one-hop data-intensive networks with star topology. They simulated a video surveillance system by continuously transmitting images to a sink node. Among other outcomes, their study determined that the Packet Error Rate for this kind of traffic, where packets have the maximum size allowed by the IEEE 802.15.4 standard, presents a significant increase as the network grows in comparison with typical WSNs with scalar sensors and short packets. In the same line, Pekhteryev *et al.* [11] performed a similar research in ZigBee networks that showed the difficulties in transmitting images over multi-hop networks. Although they only compared one-hop and two-hop schemes, they observed an important decrease in the percentage of recoverable images at the receiver for the two-hop scenario (from 2.6% to 20.5% using JPEG images, while for JPEG 2000 images the tests for the two-hop scenario could not completed due to interferences from other nodes).

There are other studies that focus on optimizing wireless performance communication in WMSNs in different ways. One of them considers cross-layer approaches to improve QoS. García-Sánchez *et al.* [12] used application-level QoS parameters to tune the MAC and physical layers of the popular IEEE 802.15.4 standard. An important increase in the maximum throughput for one-hop multimedia transmissions was achieved by means of a series of optimizations which maximized the amount of data transmitted and avoided collisions due to imperfect synchronization and other issues. In addition, the proposed solution remained compliant with the original standard. Farooq *et al.* [13] proposed another cross-layer architecture for multi-hop clustered networks where, upon congestion, nodes are requested to reduce the data transmission rate to avoid congestion. The authors used a differentiated services approach that classifies data traffic according to six different classes and where nodes producing less data and low priority data are penalized more than those producing high volumes of data and high priority data. Alanazi *et al.* [14] evaluated other QoS routing protocols for real-time WMSN applications, among which the Pheromone Termite (PT) protocol [15] offered better performance. The protocol, though, relies on both ad-hoc MAC and, more importantly, physical layers, which makes its implementation on real devices very difficult.

In video transmission, the importance of each video frame can be used to select different QoS profiles. Kandris *et al.* [16] developed a video distortion model that made it possible to predict the effect on video quality of dropping each packet. In case of congestion, typically found when transmitting multimedia flows, this model is used to selectively drop less significant packets prior to transmission, therefore improving performance at the expense of additional computing load. The authors also used a hierarchical routing protocol with asymmetrical communications, where the sink is able to transmit directly to all nodes, with no intermediary hops. Zaidi *et al.* [17] developed a multipath protocol that obtained three different paths toward a sink node and, based on Bit Error Rate and delay, selected the best of them for the transmission of the most important video frames (the I frames, explained in Section 3).

In general, multipath communication has traditionally been used to increase robustness or reliability in WMSNs. For example, the MMSPEED protocol [18] uses probabilistic multipath forwarding to control the number of paths based on the required delivery probability. In this way, depending on the packet loss rate and QoS requirements, it can send multiple copies of a same packet to ensure the delivery of data. In WMSNs, multipath can also be used to increase the throughput in data-intensive flows. Teo *et al.* [19] proposed a routing protocol, I2MR, that transmits simultaneously through two paths. In order to avoid interference between them, the protocol uses the shortest path between source and destination as the primary path and marks one- and two-hop neighbors of nodes in the primary path as the interference zone. A secondary path as well as a backup path are then constructed using nodes outside the interference zone. This protocol is intended for less energy-constrained 802.11 networks, but there are similar proposals using more energy-constrained standards. Maimour [20] developed the MR2 protocol. This protocol, which is better suited for dense WSN deployments, is also interference-aware, and constructs paths incrementally. After obtaining a new path, all of the nodes neighboring to the path, which potentially may cause interference, are notified and put in a passive mode and cannot be used for new paths. Therefore, avoiding interference and saving energy. Li *et al.* [21] proposed GEAM, a Geographic Energy-Aware Multipath routing scheme that divides the network into what the authors named “districts”. The distance between them is sufficiently large to avoid transmission interference. To send a packet, GEAM assigns the packet to a district and forwards it to the sink using the greedy algorithm. Consequently, it is necessary that each node is aware of its location. Bidai *et al.* [22] developed another multipath routing scheme for video transmission that combines proactive routing, which provides fast response to events of interest, with reactive routing. In this way, upon the detection of an event, the transmission of data packets to the sink starts immediately and, simultaneously, a route discovery phase begins where new paths to the sink are discovered. These paths are selected according to a metric defined by the authors that estimates their amount of interference. Simulation results demonstrate that selecting the less interfering path can increase throughput and decrease packet loss.

Table 1 shows the main contributions of the abovementioned proposals, as well as other features. As it can be observed, many of the proposals use either the IEEE 802.11 standard or the non-beacon mode of the IEEE 802.15.4 standard, which does not define how duty-cycling should be carried out. The 802.11 standard allows higher data transmission bit rates, therefore allowing for higher data throughput. However, it is not well suited for WSN because of its power consumption requirements. Similarly, 802.15.4 without a sleep/wake-up schedule, *i.e.*, duty-cycle, draws a considerably higher amount of power, albeit not as high as with 802.11. In the case of short-range transmissions, such as the typically used in WSNs, transmission power is lower than the power spent in reception mode [23], therefore the most effective measure to limit power consumption is to make devices remain as much time as possible in low power (idle) states. For this same reason, battery-powered ZigBee-based solutions, where the non-beacon enabled mode is used, are known to have shorter network lifetimes simply because it forces all nodes with routing capability to be always on, with the subsequent battery drain. Finally, other routing schemes used in the discussed proposals exhibit other concerns. Hierarchical routing tend to overload nodes in the top layers of the hierarchy, which are closer to the sink, and, a priori, have more complex route repair procedures (*i.e.*, it takes some time to adapt to node failures, provided that the protocol is designed to perform route repair). Multipath schemes may overload some nodes if the path selection criteria lead to always choosing the same paths, potentially affecting network lifetime.

As stated before, the purpose of this work is to demonstrate the feasibility of video transmission using the IEEE 802.15.5 mesh standard, which is designed to create highly robust and reliable communication links. This standard allows for a very low power operation by defining how nodes should alternate periods of activity and inactivity to save energy and how these same nodes should coordinate with one another for a reliable transmission of data. This work is also intended to provide the scientific community with simulation tools for further evaluation of new protocols and improvements for WMSNs.

## 3. Technological Background

The next sections describe the details of the technologies related to this paper that are more relevant to the simulations performed. First, the main parameters involved in H.264 video encoding are explained. Second, the low-rate part of the IEEE 802.15.5 standard is introduced.

### 3.1. H.264 Video Encoding

As already stated, the transmission of video in low-power WSNs is challenging due to their inherent constraints. The transmission of large amounts of data poses additional problems such as contention for medium access, which increases jitter and further decreases throughput. It is clear that in order to cope with these restrictions video compression is needed. As an example, a typical QCIF sequence (176 × 144 pixels) at 30 frames per second requires almost 6 Mbps to be transmitted uncompressed via streaming. However, the nominal transmission rate of IEEE 802.15.4-compliant devices, such as MICAz [7] or TelosB [24], is 250 kbps when operating in the 2.4 GHz ISM band.

H.264 [25], also known as MPEG-4 Part 10 or MPEG-4 Advanced Video Coding (AVC), is one of the most popular standards for video encoding. It is about 1.5 to 2 times more efficient than MPEG-4 (Part 2) encoding, resulting in smaller compressed files. This is crucial for WMSNs, which are severely constrained by the transmission rate and energy consumption of nodes. The main benefit arising from the use of H.264 in WMSNs is the reduction in the number of data messages necessary for transmitting a video frame. This further helps reduce the network congestion and prolong the nodes lifetime. As a drawback, H.264 encoding requires more energy at the source node. However, according to [26], the use of H.264 results in a better lifetime in networks with at least five hops between the sensor nodes and the data sink and also in smaller networks with more complex video contents or more stringent video quality requirements. A more detailed study about energy spent in video encoding can be found in [27].

Several encoding parameters used by H.264 (GOP size, Quantization Parameter and CRF mode), which will be referred to subsequently, are described next.

The Group of Pictures (GOP) structure specifies the order in which different types of frames are arranged in inter-frame encoding techniques. H.264 uses three different types of frames:

- I-frames (intra-coded frames). I-frames are encoded independently of all other frames. They are the largest frames and provide the best quality, but they are also the least efficient from a compression perspective.
- P-frames (predictive-coded frames). P-frames reference redundant information contained in previous frames (I or P frames). Therefore, they contain a lesser amount of information.
- B-frames (bidirectional predictive-coded frames). B-frames can reference both previous and future frames. From a compression perspective, they are the most efficient frames.

Encoders usually require the GOP size as a parameter, which is the number of frames in a GOP structure. Since a GOP only contains an I-frame at the beginning of the structure, this parameter sets the separation between two I-frames.

For each frame, the Quantization Parameter (QP) regulates how much spatial detail is saved or discarded. In this paper, rather than using a fixed QP for each frame, the Constant Rate Factor (CRF) mode is used instead. With this mode, the encoder tries to achieve a constant perceptual quality level for all frames by adjusting the QP according to the characteristics of each frame. That is, it uses higher compression rates for frames with high motion, as the loss of detail is harder to notice in moving objects. A CRF parameter, ranging from 0 to 51, is defined to control the desired quality of the video, where lower values result in better quality and higher values in more compressed video sequences.

**Figure 1:**
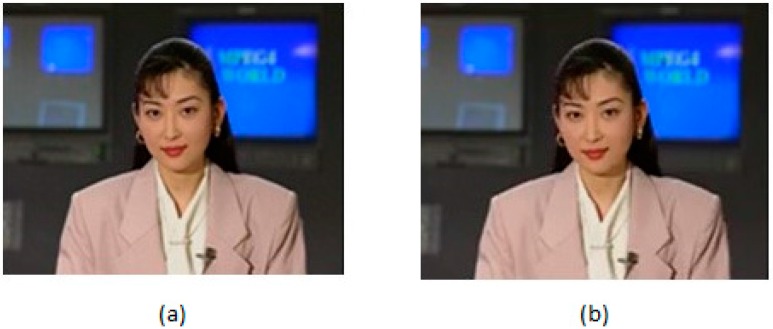
(**a**) Akiyo QCIF, CRF = 10; (**b**) Akiyo QCIF, CRF = 24.

In this work, the value of the CRF has been set to 24 since this offers a balance between video quality and compression. In Table 2 different metrics are compared for CRF values of 10 and 24 using the same video sequence. It can be observed that a value of 24 decreases the bitrate of the compressed video and, consequently, the transmission time over a 6 × 6 grid mesh network. The drop in quality of the video is very small, as can be observed by comparing Figure 1.

### 3.2. IEEE 802.15.5 Low Rate

IEEE 802.15.4 standard defines Physical (PHY) and Medium Access Control (MAC) specifications for Low-Rate Wireless Personal Networks (LR-WPAN). This standard does not define the network layer and, therefore, does not offer routing capabilities by itself. Consequently, with the aim of providing an efficient multi-hop scheme, the IEEE 802.15.5 standard emerged in 2009. This new standard has a low-rate part, which will be the focus of the paper, consisting of a set of recommendations, which, according to the standard, provide an architectural framework that enables low-power, low-rate WPAN devices to promote interoperable, stable, and scalable wireless mesh topologies [28].

The objective of the standard is to support features such as unicast, multicast and reliable broadcast over multi-hop mesh links, synchronous and asynchronous communication for power saving, trace route functionality and portability of end devices [28]. In addition, being a mesh standard, it provides route redundancy, which enhances network reliability. Another key feature of the standard is that it fosters simplicity. There is no need for route discovery, reducing communication overhead, or for storing routing tables for all possible destinations. One of its main advantages over other mesh networking approaches is that it has a very similar set of service access points to the 802.15.4 MAC sublayer and, therefore, migration from non-mesh IEEE 802.15.4 networks to mesh networks is a straightforward process. In this regard, IEEE 802.15.5 aims at standardizing mesh networking over IEEE 802.15.4.

**Figure 2:**
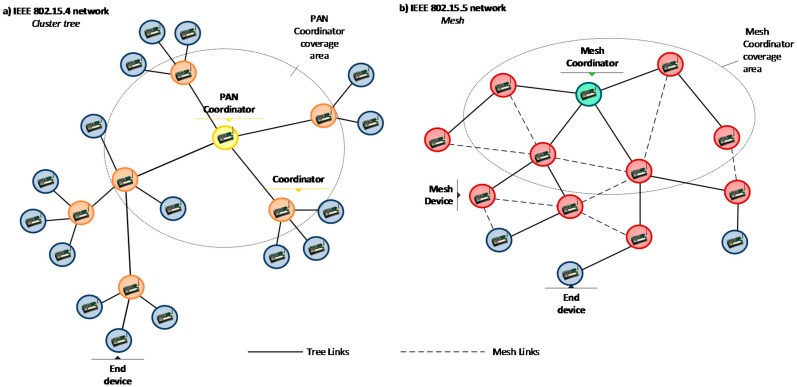
Topology examples. (**a**) IEEE 802.15.4 cluster tree; (**b**) IEEE 802.15.5 topology.

Figure 2 shows a comparison of the topologies of an IEEE 802.15.5 network with a cluster-tree IEEE 802.15.4 network. The main difference lies in the mesh links (represented by dashed lines), which connect any node with any neighbor node within communication range. The types of device defined in an IEEE 802.15.5 network are also shown in the Figure. A *mesh coordinator* is the root of the logical tree of the mesh network. It creates and manages the mesh network, and may also serve as the reference clock when network-wide synchronization is required. *Mesh devices* are responsible for intelligently relaying sensor data toward their intended destination. Finally, *end devices* are generally nodes with in-built sensors and do not include mesh routing capability.

The establishment of the network topology is key to the operation of IEEE 802.15.5. During this process, logic addresses are assigned to nodes according to their position in the network. By binding logic addresses to the network topology, the Tunneled Direct Link Setup (TDLS) routing protocol [29] suggested by the IEEE 802.15.5 standard can forward packets without performing route discovery, eliminating the associated latency and reducing communication overhead as well as avoiding the need for explicit route repair. This protocol, which measures route quality in terms of hop count, balances traffic among all the most suitable paths.

#### 3.2.1. Energy-Saving Mechanisms

In other WSNs solutions such as Zigbee, nodes’ radios are forced to remain on listening for any incoming transmissions. This involves a considerable power consumption and, consequently, a reduction of the lifetime of nodes. To cope with this problem, the low-rate part of the IEEE 802.15.5 standard has two energy-saving modes, called Asynchronous Energy Saving (ASES) and Synchronous Energy Saving (SES). These modes define the duty cycles of nodes, that is, the time during which their transceivers are active or inactive. ASES and SES are intended, respectively, for asynchronous and synchronous communications.

**Figure 3:**
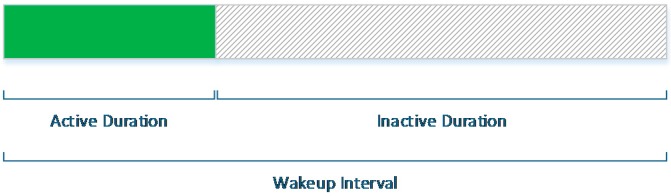
Time structure of IEEE 802.15.5 energy-saving mechanisms.

The mechanisms proposed by the IEEE 802.15.5 standard, the ASES and the SES mechanisms, have some similarities regarding the time structure in which they schedule active and inactive periods of the radio. Figure 3 shows this duty-cycle schedule for every node. The *wakeup interval* (WI) refers to the length of one duty cycle. This cycle is divided into an *active duration* (AD), where the radio transceiver is always on, followed by an *inactive duration* (ID), where the radio, MCU and sensors enter low power modes (if available) to save energy.

The length of a *wakeup interval* is defined by the *wakeup order* (WO) parameter according to the following expression:

(1) $$WI=\;meshBaseActiveDuration\;\times 2^{WO}\;0\leq WO\leq 14$$

Similarly, the *active order* (AO) parameter controls the *active duration*:

(2) $$AD=meshBaseActiveDuration\times 2^{AO},\;0\leq AO\leq \;WO\leq 14$$

where *meshBaseActiveDuration* is a constant defined by the IEEE 802.15.5 standard with a 5 ms default value.

#### 3.2.2. Asynchronous Energy Saving (ASES)

In the ASES mode, nodes are configured with the same *wakeup interval* but may have different *active durations*. Also, there is no synchronization among the *active durations* of network nodes. In order to enable communications, one or some of the nodes, depending on the case, have to delay their *active durations*. For instance, unicast communication requires that every potential receiver periodically sends a broadcast frame advertising its *active duration*. When any other node wants to transmit, upon receiving these broadcast frames, it sends its pending data during the *active duration* of the receiver using CSMA/CA (Carrier Sense Multiple Access with Collision Avoidance) for medium access. For further details about the operation of the ASES mode, the following references give more insight [28,30].

The ASES mode is intended for networks with low data sampling and data transmission rate requirements and it supports node mobility. However, it is not appropriate for video transmission because it is not able to provide an adequate QoS for more intensive data flows.

#### 3.2.3. Synchronous Energy Saving (SES)

Unlike ASES, the Synchronous Energy Saving (SES) mechanism is intended for static networks. It is based on a precise synchronization mechanism of the *active* and *inactive durations* of the nodes, which potentially decreases delays in packet transmissions. It is well-suited for delay-sensitive applications, ensuring quick reporting of events of monitoring interest.

SES defines two transmission methods, namely the *contention-based method* and the *reservation-based method*. In the *contention-based method*, devices can only carry out data transmission within the *active duration*, competing with other nodes for medium access. In the *reservation-based method*, nodes can transmit data within the *inactive duration* when other nodes are in sleep mode. In this way, it is possible to take advantage of the available transmission bandwidth to relay data, making it potentially suitable for reliable transmission of multimedia data.

In the *reservation-based method*, the *inactive duration* is divided into slots of a fixed length. Every time a node wants to transmit, it sends a *reservation request* message during the *active duration*, competing with other nodes for accessing the medium through the CSMA-CA mechanism. If the reservation succeeds, a slot in the *inactive period* is assigned to transmit to the next hop in the source-destination route. The reservation process is repeated for each hop in the route during the *active duration*. Once the *inactive duration* starts, all the successfully reserved slots are used to transmit packets from the source to the following nodes in the route. If the *active duration* has ended and there are no slots reserved for all the hops in the route, the reservation for the remaining hops of the route will continue in the next *active duration*.

**Figure 4:**
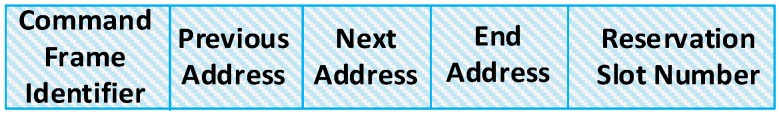
Format of the reservation request message.

The format of the *reservation request* message can be observed in Figure 4. As the names of the fields suggest, the *end address* is the address of the destination or sink node, the *next address* is the address of the next hop of the route, selected by the TDLS routing protocol, and the *reservation slot number* identifies the requested slot. The purpose of the *previous address* field is to save bandwidth, since the same message used to request the reservation of a slot can also be used to inform the previous node that its reservation was accepted.

**Figure 5:**
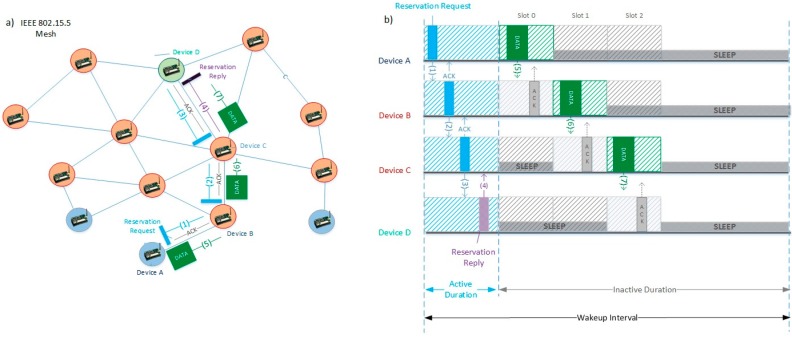
Data transmission in SES. (**a**) Topological view; (**b**) Time structure view.

As an example of the *reservation*-*based* method, Figure 5 illustrates a scenario where node/device A transmits to device D through a route consisting of devices B, C and D. First, A sends a *reservation request* message (identified in the figure by label 1) setting the *end address* to D, the *next address* to B and the *reservation slot* to zero (first slot). The message is sent to the broadcast address and received by all neighbor nodes but only B processes it. In a second step, B sends a *reservation request* (label 2) for the second slot to C (*next address* of the message) and acknowledges A that it has successfully reserved the first slot by means of the *previous address* field. Next, device C sends a reservation request (label 3) for the third slot to D. Finally, D sends a *reservation reply* message (label 4) to C confirming the allocation of the third slot. After the reservation in the *active duration*, the data transmission starts in the *inactive duration*, each device using the slot that was assigned (labels 5–7) and receiving acknowledgement frames from the receivers during the same slot. This example shows, as mentioned previously, how the *reservation-based method* can forward a data frame from the source to the destination, using a single *inactive duration*.

## 4. Simulation

### 4.1. Simulation Environment

In order to carry out the simulation of multimedia transmission in an IEEE 802.15.5 mesh network, ns-2 simulator and EvalVid software are used. ns-2 [31] is a popular discrete-event network simulator, which provides support for assessing many different protocols and network technologies, including IEEE 802.15.4.

The other component used for the simulations, EvalVid, is a tool-set for evaluating the quality of video transmitted over a real or simulated network [32]. It can be used to measure several QoS metrics of the underlying communication network such as packet loss, delay, jitter and standard video quality metrics like PSNR. Unlike other available tools, EvalVid works properly even if there are packet drops, making it suitable for unreliable networks such as WSN.

**Figure 6:**
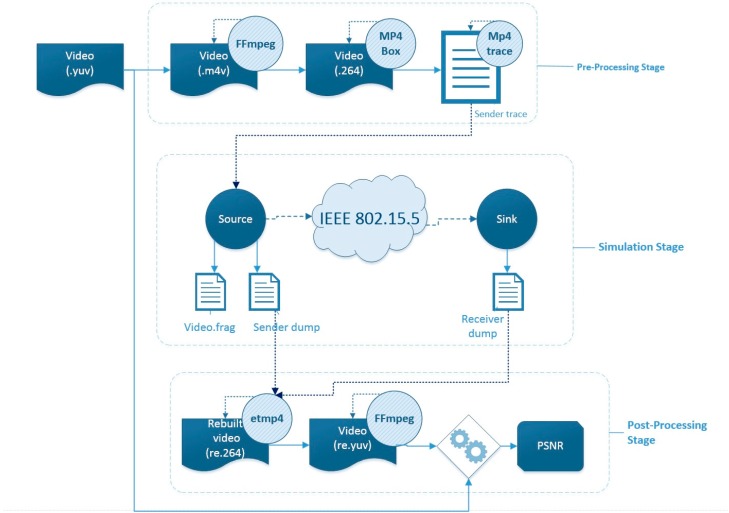
Simulation workflow.

In order to provide researchers with an integrated multimedia tool for simulating WSNs with mesh topology, a new module was added to ns-2. This new module, accessible via [33], includes the IEEE 802.15.5 SES mode and its integration with EvalVid. In order to perform the simulation experiments, the video is encoded and some information on how to fragment each video frame for its transmission in an IEEE 802.15.5 network is generated. This information is passed to ns-2, which simulates the transmission of packets. It is also used by EvalVid, in conjunction with data about the arrival of packets, to calculate the aforementioned QoS metrics of interest. Figure 6 shows the steps involved in the simulation and detailed in the next paragraph.

The original file stores information using the YUV (Y luminance component, U and V chrominance components) color space as it allows for greater compression than Red, Green, Blue (RGB). Since the human eye has little spatial sensitivity to color, the bandwidth of the UV chrominance channels can be considerably reduced with little impact on visual perception. The information in this file is sequentially processed by the *FFmpeg* tool to encode the video and the *MP4Box* tool to create an H.264 flow with video frames and a *hint track*. This track describes how to fragment each video frame that is going to be transmitted. From the resulting file, EvalVid’s *MP4trace* tool creates a text file, the *sender trace*, which contains the information about every frame shown in Table 3.

The information of the *sender trace* file is used for generating the *video1.frag* file (Table 4) in order to adjust the file to the requirements of the ns-2 framework and to perform the simulation. This file has all the necessary information to allow a modified User Datagram Protocol (UDP) agent to transmit video fragments at the appropriate time. This agent will be in charge of passing the fragments to the IEEE 802.15.5 mesh layer for their transmission in the simulated network, where packet delays or losses are detected and computed. The agent, in addition, stores the generation time of each packet in a file known as the *sender dump*. Upon reception, another modified UDP agent generates another file, the *receiver dump*, with the arrival times of each packet.

Finally, EvalVid, by means of the ET (Evaluate Trace, *etmp4*) tool, makes use of the encoded video and the *sender trace*, *sender dump* and *receiver dump* files to generate reports with the most relevant metrics: latency, jitter and number of packets and video frames lost. In addition, after decoding the video with the *ffmpeg* tool, the PSNR quality metric can also be obtained.

### 4.2. Simulation Scenarios

Simulations consisted of the transmission of commonly used video sequences [34]: Akiyo QCIF, Foreman QCIF and Mobile QCIF. These video sequences were selected because of their different motion, sorted in ascending order according to their motion. Five simulation scenarios were chosen with different numbers of nodes. In the scenarios, nodes were arranged in a regular grid layout, ranging from a 2 × 2 network to a 6 × 6 network. For each scenario, simulations were performed for various configurations of the WO and AO parameters, resulting in different lengths of the *active duration*, the *inactive duration* and the number of transmission slots in the *inactive duration*. Table 5 shows the WO–AO combinations used and the length of each period of the SES time structure according to Equations (1) and (2).

The length of each slot within the *inactive duration* was chosen to be 10 ms [35]. This was regarded as sufficient for carrying out the transmission of a single data message between two consecutive nodes.

Table 6 shows the set of network simulation parameters common to all the simulations performed. With regard to the video encoding parameters, the GOP size was set to 25, CRF to 24 and frames per second (FPS) to 25.

## 5. Performance Evaluation

**Figure 7:**
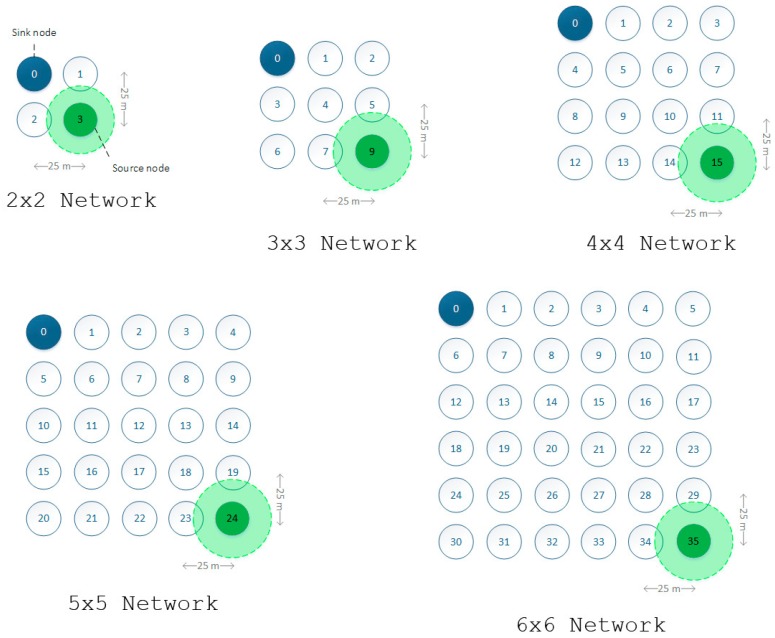
Simulation scenarios.

The simulation results for every scenario (Figure 7) are presented below. Different metrics of interest have been plotted, including latency, jitter, throughput, message delivery ratio, power consumption/network lifetime and PSNR. These metrics are commonly assessed in other studies related to multimedia traffic or WSNs. It should be noted that, for all scenarios, power consumption and lifetime were computed for node 1, as will be explained later in the text. To conclude this section, a scalability study is also included. It is intended to show the impact of the aforementioned metrics in networks composed of a large number of nodes and different node densities.

### 5.1. Latency

Latency has been computed as the average time between the generation of data messages and their delivery to the destination node (*i.e.*, data sink). This includes the time dedicated by all the intermediate nodes belonging to the source–destination path for accomplishing tasks such as the data processing, temporary storage in the node's memory or transmission/reception procedures, among others.

**Figure 8:**
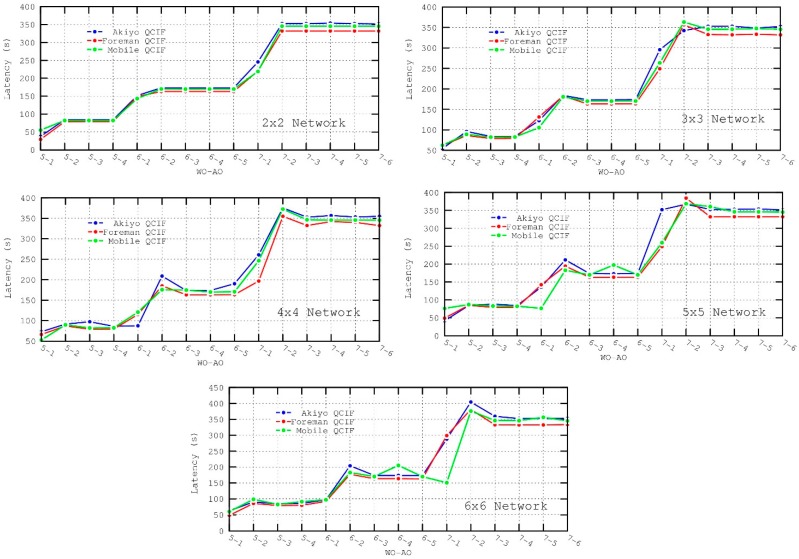
Latency metric for the different scenarios.

The latency for all the packets that have successfully reached the destination is shown in Figure 8. It can be observed that the latency shows a considerable variation for different values of WO. It should be noted that while the generation rate remains constant at 53 kbps, only one packet is transmitted per *wakeup interval*, whose length is defined by this parameter. Consequently, as WO (and subsequently the *wakeup interval*) increases, the capacity of the network to accommodate data traffic decreases and packets are stored in the queue of the first node waiting for transmission, therefore increasing the latency measured. Some simulations were also performed transmitting two packets per *wakeup interval*, but the obtained performance was really poor. For example, for a 6 × 6 configuration, an increase in the latency of around 50% was measured. In this case, increasing the amount of data sent led to a higher contention for medium access, which worsened the performance of the network.

**Figure 9:**
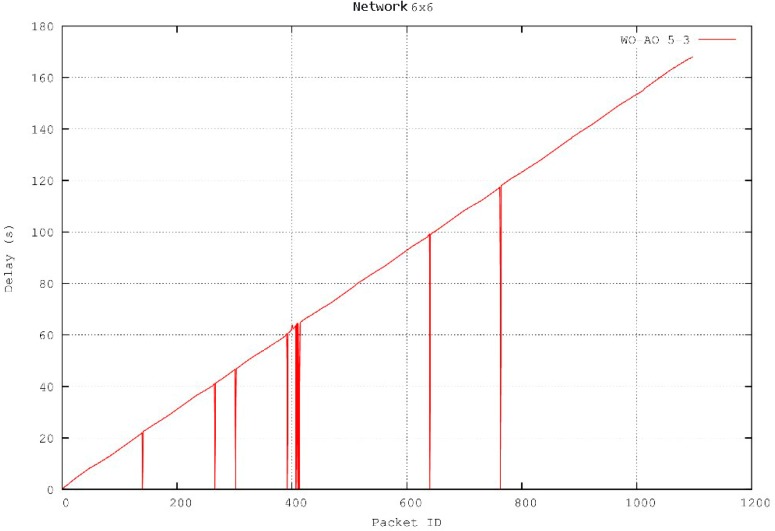
Packet latency.

Under these circumstances, for any given WO–AO configuration, the latency increases linearly with respect to the number of transmitted packets (see Figure 9) because of the packet service rate of the network (one packet per WI). The figure shows the evolution of the latency calculated after the arrival of each packet for a 6 × 6 network with a 5–3 (WO–AO) configuration. It has to be noted that latency values of zero in the figure represent packet loss.

In Figure 8 there is a case that requires further explanation, specifically for AO = 1, where the latency measured is smaller. However, these low values are obtained because of high contention for medium access and the resulting packet loss. These figures must be analyzed in conjunction with the Message Delivery Ratio figures. For AO = 1, it can be observed that packet loss increases considerably since the *active duration* is too short to successfully reserve all transmission slots. In particular, as the congestion of the network grows with time, most of the packets that are successfully received correspond to the first packets sent, which have a lower latency. On the contrary, packets sent subsequently, which have a higher latency as shown in Figure 9, have a higher loss probability. Since the displayed latency is averaged over all the successfully received packets, this results in a deceptively short latency.

Besides, it can be observed that, for a fixed value of WO, the latency remains almost constant in the interval 3 ≤ AO < WO. Thus, a further increase of the *active duration* will not have noticeable effects on the latency. This holds true even for AO = WO − 1, where the *inactive duration* will still be long enough. According to Table 5, for AO = WO − 1, the *inactive duration* is as big as the active duration and thus adequate for transmission.

In conclusion, for a fixed value of WO, the latency remains almost constant except for the two following cases:

- For AO = 1 the *active duration* is too short and, given that the contention for the medium is too high, few nodes are able to reserve a slot for transmission and soon the network experiences congestion collapse. Packets are stored in queues (of infinite size) and many of them, due to the failure of the CSMA-CA mechanism (no successful transmission after the maximum number of retries), are discarded and will not reach the destination. The latency measured corresponds to the few packets that are successfully received.
- For AO = 2, the contention is still high (the *inactive duration* is not long enough yet) but packet loss is not as important as in the previous case (though still present). Therefore, the average delay represented is higher since the final packets (with higher latency) experience less congestion than for AO = 1. However, the latency measured is also slightly higher than for AO values greater than 2 because of the network congestion, as the transmission of packets may require more than one *wakeup interval*.

### 5.2. Inter-Packet Arrival Time

Inter-packet arrival time refers to the time that elapses from the receipt of a packet at the data sink to the instant at which the next packet is received.

According to the operation of the SES *reservation-based method*, it can be expected that the time between the receipt of two consecutive packets at the destination node is determined by the time between slots that one particular node has available for transmission. This time is set by the length of the *wakeup interval*, which, in turn, depends on the WO parameter. The theoretical inter-packet arrival time is:

(3) $$WI=meshBaseActiveDuration\times 2^{WO}=5\;ms\times \;2^{5}=160\;ms,\;if\;WO=5$$

(4) $$WI=meshBaseActiveDuration\times 2^{WO}=5\;ms\times 2^{6}=320\;ms,\;if\;WO=6$$

(5) $$WI=meshBaseActiveDuration\times 2^{WO}=5\;ms\times 2^{7}=640\;ms,\;if\;WO=7$$

(6) $${\bar{it}}_{P_{n}}=\{\begin{array}{c} 160\;ms,\;WO=5\\ 320\;ms,\;WO=6\\ 640\;ms,\;WO=7 \end{array}$$

**Figure 10:**
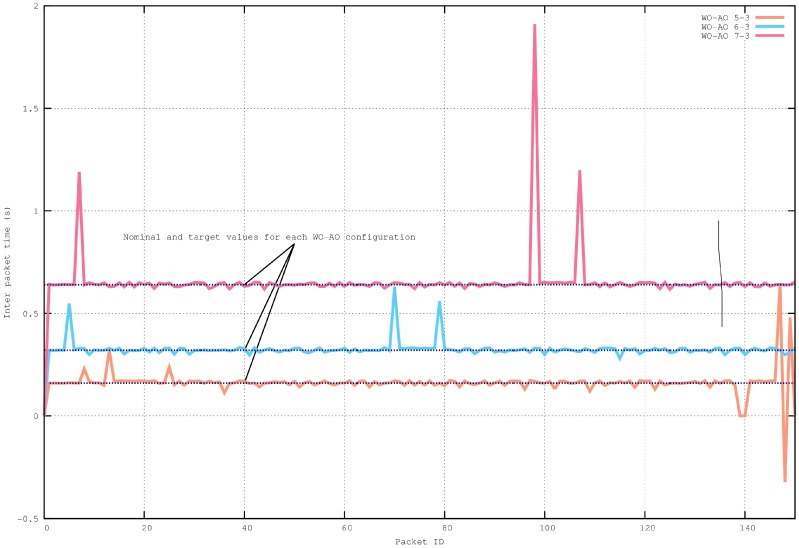
Inter-packet arrival times for a 6 × 6 network.

Increasing the WO by one doubles the inter-packet arrival time, causing a decrease in throughput. This has been tested through simulations of a 6 × 6 network with WO–AO configurations in Figure 10.

It can be observed that the obtained measurements are consistent with the theoretical values, with values around 0.16 s for WO = 5, 0.32 s for WO = 6 and 0.64 s for WO = 7. There are, however, small variations in the inter-packet arrival times, also known as jitter, which will be discussed in the next section. These variations are mainly due to the imperfect synchronization process performed in the SES mechanism and the CSMA-CA mechanism. In addition, larger variations, also caused by the CSMA-CA mechanism, may indicate packet delays through several *wakeup intervals* and, when packets arrive out of order, negative values may be found.

### 5.3. Jitter

Jitter is calculated as the variation in the inter-packet time caused by network congestion, packet loss, loss of synchronization, or by the use of multiple paths from source to destination. It must be noted that due to the use of the TDLS routing protocol, which by default selects the shortest paths between source and destination and performs load balancing across them, all the paths used for data forwarding will have the same number of hops. Therefore, in spite of the use of multiple paths, all the packets will traverse the same number of hops. Consequently, the jitter will not be affected.

**Figure 11:**
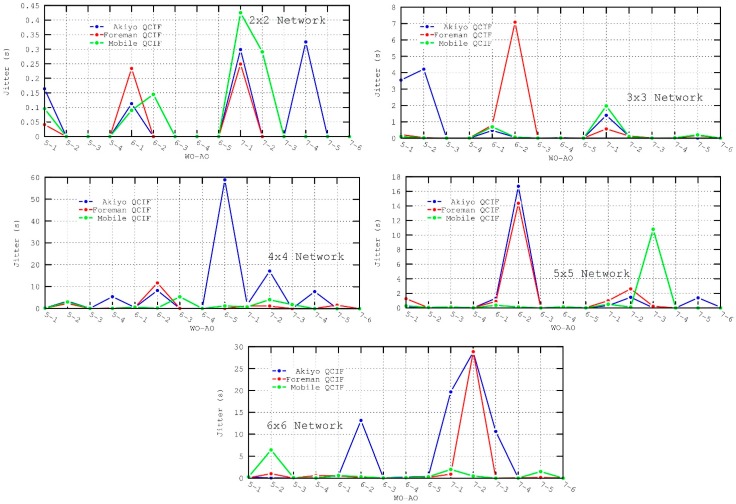
Jitter metric for the different scenarios.

However, there are situations where jitter is more likely to be affected. Analyzing Figure 11, when AO=1 or AO=2 the jitter increases since the *active duration* is insufficient to perform all slot reservations, resulting in more congestion and consequently in more unpredictable inter-packet arrival times.

### 5.4. Throughput and Message Delivery Ratio

Throughput is defined as the amount of raw information (header and payload) received in a given period. From this definition, it is clear that throughput is directly related to the *message delivery ratio metric*, that is, to the rate of successfully received messages. Consequently, these two metrics are discussed below jointly.

In order to evaluate the *message delivery ratio*, an entry is created in a text file for each message transmitted or received, as explained before, letting EvalVid check the number of packets sent and received.

It should be noted that UDP is used at the transport layer over the IEEE 802.15.5 mesh network layer and IEEE 802.15.4 MAC and PHY layers. Therefore, if a packet does not arrive at the destination node for whatever reason, the packet will not be retransmitted.

Since the size of queues has been assumed to be infinite, it can be deduced that packet losses are not related to the size of buffers. Consequently, packet losses are mainly caused by the occupation of the channel and the failure when accessing the medium through the CSMA-CA mechanism (no successful transmission after the maximum number of retries). In addition to existing medium contention, the exposed node problem and the hidden nodes problems also affect transmissions in the *active duration*, since CSMA-CA does not avoid collisions by hidden nodes or unnecessary backoff delays by exposed nodes [30]. The exposed node problem prevents a sender from transmitting to a receiver if the sender detects a signal from another node, even if that signal is not interfering at the receiver’s location. This increases the probability of CSMA failures. Besides, even if a node manages to transmit a message, it may be lost due to the hidden node problem. In this regard, the reservation process that takes place during the *active duration* will be affected by both primary (two hidden nodes transmitting to the same receiver) and secondary (receiver in range of another node transmitting to a different node) collisions. On the other hand, the *inactive duration* will be affected to a lesser extent by the hidden terminal problem, as discussed in Section 5.6.

**Figure 12:**
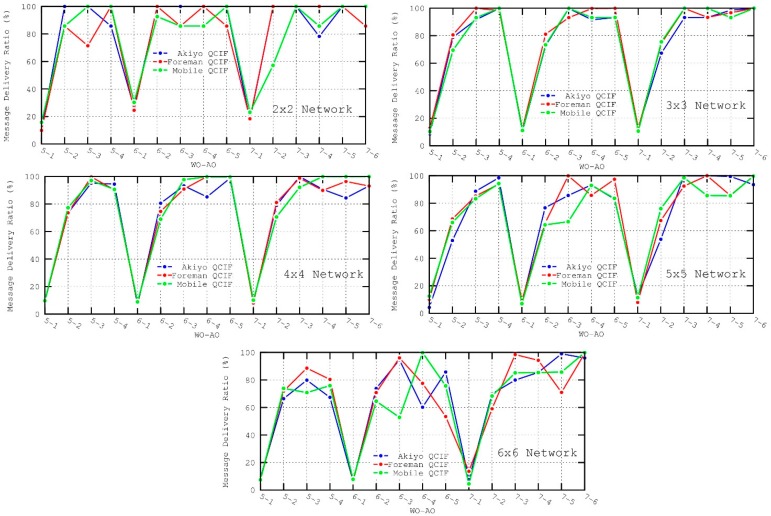
Throughput metric for the different scenarios.

**Figure 13:**
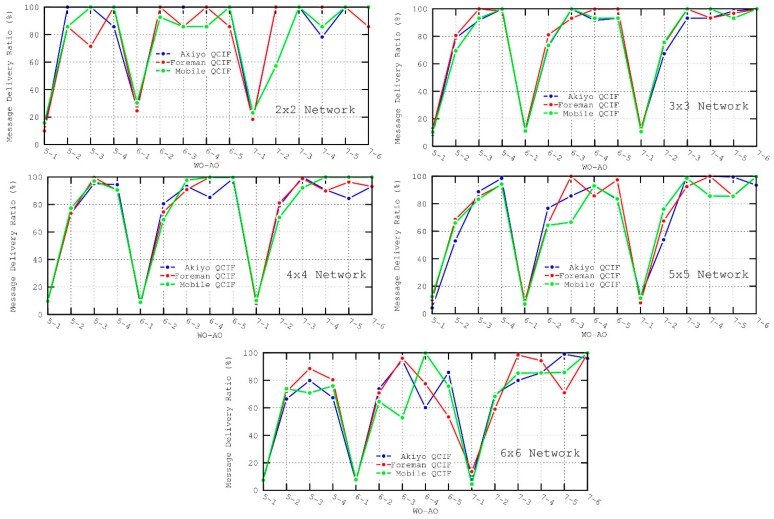
Message delivery ratio metric for the different scenarios.

As observed in Figure 12 and Figure 13, AO values of 1 and, to a lesser degree, 2 present the worst performance in both the throughput and message delivery ratio. In addition, throughput is also affected by the selection of WO values, since it determines the separation between consecutive packets sent. That is, a longer *inactive period* (or WO values) implies lower throughput. In particular, the best results for the throughput metric are obtained for 5–3 (WO–AO) configuration. In this case, the lengths of the *Wakeup Interval* and Active Duration are found to be suitable for forwarding data messages without incurring long node delays.

### 5.5. Power Consumption and Lifetime

The SES mechanism provides an efficient energy-saving mechanism because the devices are sleeping most of the time, turning on their transceivers during short periods of time for transmitting or receiving video packets. In addition, according to the type of device in an IEEE 802.15.5 network (*i.e.*, whether the node acts as *mesh coordinator*, *mesh or end device*), it can be intuitively deduced which of them will have a higher power consumption during the *inactive period* of the *SES reservation-based* method:

- *Mesh coordinator*: Most of its power consumption is due to the continuous performance of synchronization tasks [28], which are out of the scope of this paper. Regarding data transmission power consumption, which is the focus of this section, the mesh coordinator will not have to perform data transmission and the only significant consumption is from packet reception during the allocated slots (it changes the radio transceiver state from *sleep* to *listening*).
- *End device*: This type of device corresponds to the source node that sends packets continuously through the network. Therefore, its power is consumed during the transmission in one slot per SES time structure, that is, one slot for each *wakeup interval*.
- *Mesh device (router)*: Unlike the other two types of nodes, mesh devices require two consecutive slots of the *inactive period*, one for receiving from the previous node and another for transmission to the next node. Consequently, they are the devices that have the highest power consumption.

On the other hand, all nodes have additional power consumption during the whole *active duration* since their transceivers are either in the *listening* state or in the *transmit* state. Consequently, the *active duration* affects to a greater extent the power consumption metric than the *inactive duration*, where nodes remain asleep for most of the time (their state is switched only to perform the scheduled transmissions/receptions in the reserved slots).

**Figure 14:**
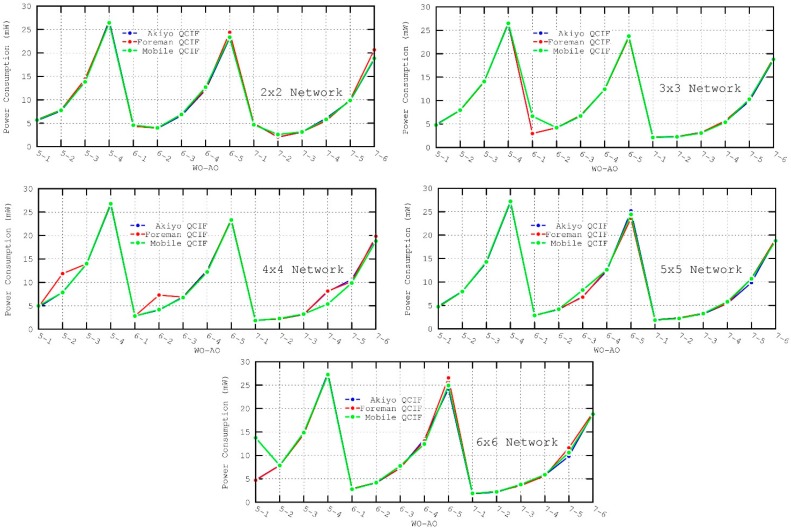
Power Consumption metric for the different scenarios.

**Figure 15:**
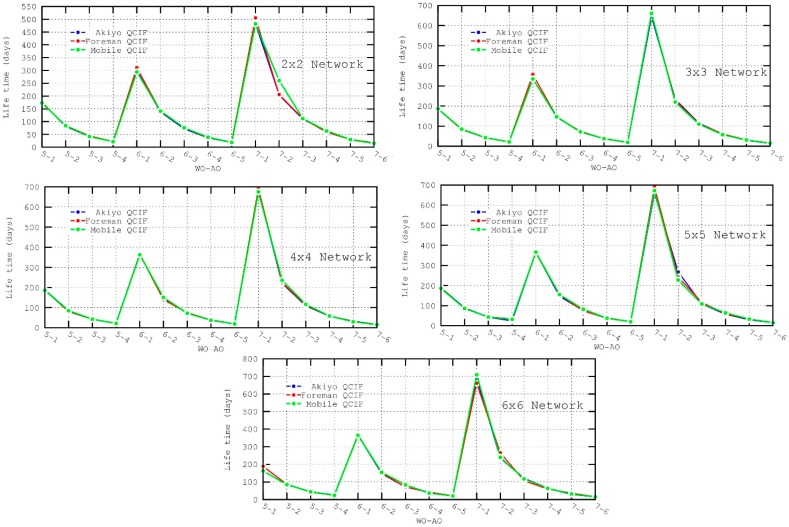
Lifetime metric for the different scenarios.

It can be observed in all the evaluated scenarios—Figure 14 and Figure 15—how the AO has a considerable impact on power consumption (regardless of the WO, nodes configured with a low AO value consume less energy). It should be noted that both power consumption and lifetime metrics were calculated for node 1, which has a high power consumption as it is a mesh device and it is located close to the destination node where all the paths generated by the TDLS algorithm converge.

### 5.6. Peak Signal-to-Noise Ratio

The most widespread method to determine the quality of a video sequence is the *Peak Signal-to-Noise Ratio* (PSNR), which represents the ratio between the maximum possible power of a signal and the power of the distorting noise that affects the fidelity of its representation. Because many signals have a wide dynamic range, PSNR values are usually expressed in logarithmic scale.

In order to compute the PSNR, the *Mean Squared Error* (MSE) of each frame must first be obtained. The MSE represents the cumulative squared error between the compressed frame and the original frame. For a monochrome image, it is calculated as:

(7) $$MSE=\frac{1}{M*N}\overset{M-1}{\underset{i=0}{\sum }}\overset{N-1}{\underset{j=0}{\sum }}{\left\|I\left(i,j\right)-K\left(i,j\right)\right\|}^{2}$$

where I(i,j) corresponds to the value of the (i,j) pixel of the transmitted frame and K(i,j) is the value of the same pixel in the received frame.

The PSNR is defined as:

(8) $$PSNR=10*\log_{10}\left(\frac{MAX^{2}}{MSE}\right)=20*\log_{10}\left(\frac{MAX}{\sqrt{MSE}}\right)$$

where *MAX* denotes the maximum possible pixel value of the image. If B bits are used per pixel (luminance component), *MAX* = 2^B^ − 1.

Considering a video sequence composed of a series of frames, the MSE and PSNR are calculated for each of the frames and the PSNR of the complete video sequence is the mean value of the PNSR of the frames.

EvalVid uses the PSNR as an evaluation metric of the quality of compressed videos. When the difference between the frames of an original video sequence and the frames of the received and decoded video sequence is high, the PSNR is low. Therefore, higher values of PSNR denote better quality of the video. Acceptable values for wireless communication systems are considered to be about 20 dB to 25 dB.

On the other hand, for the subjective evaluation of video quality, the MOS (*Mean Opinion Score*) [36] is usually employed. This technique is based on the perceived quality from the users’ perspective of the received video (or any other kind of signal), giving a score to video sequences from 1 (worst) to 5 (best). In [37] a possible mapping between PNSR and MOS values is provided. The conversion between the two metrics can be seen in Table 7.

**Figure 16:**
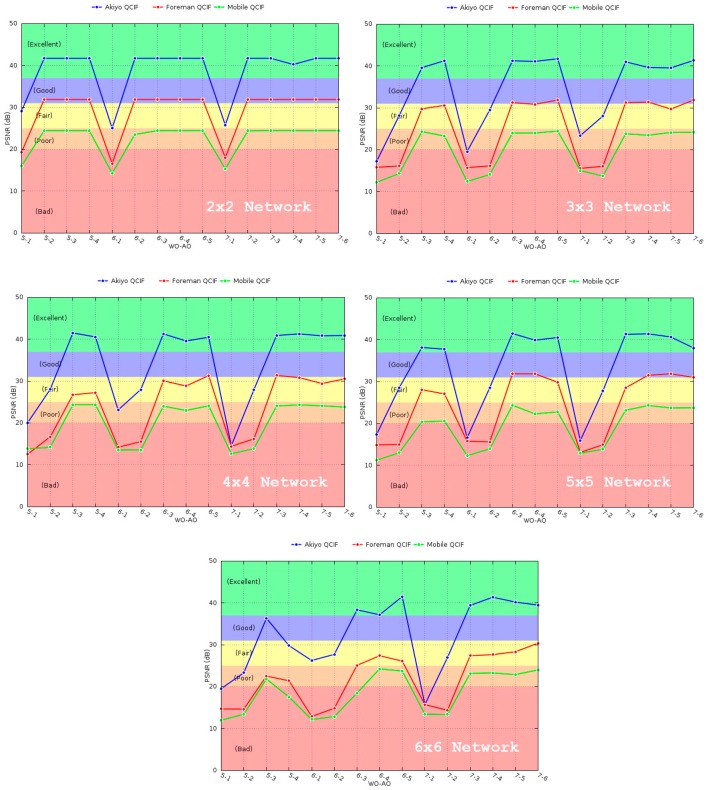
PSNR metric for the different scenarios.

Above, in Figure 16, the results of the PSNR for each of the simulated scenarios are shown. In view of the presented results, a degradation in the quality of the video can be observed as the network grows. Besides, the values of WO seem to have some impact for larger networks, 5 × 5 and 6 × 6, but not for small networks. In particular, the PSNR value deteriorates for lower WO values due to the hidden terminal problem. With fixed AO values and therefore fixed *active duration* and contention for medium access, the only reason for this drop in performance is packet loss in the *inactive duration*. Since there is no contention for medium access during this period, packet loss is due to the hidden terminal problem. More particularly, only secondary collisions from hidden nodes’ transmissions can take place, whereas primary collisions cannot happen because of the slot reservation process performed in the *active duration*.

As a consequence of the above-mentioned, the size of the network has to be taken into account for the appropriate selection of the WO, finding a balance between the quality of the video and throughput (high WO values reduce the throughput considerably).

With respect to the AO parameter, the worst PSNR values are obtained for AO = 1 and AO = 2, since the large amount of packet losses distorts the quality of the decoded video. For 3 ≤ AO < WO there is little variation in the PSNR, implying a good or excellent video reproduction.

Comparing the three different video sequences, it can be easily deduced that, regardless of the network size, the PSNR gets worse for sequences with higher motion. However, this can be misleading, since the drop in quality is not as high as it may seem. This is due to the use of the CRF encoding method. CRF compresses different frames by different amounts, taking the motion into account. Therefore, frames with higher motion will have a greater compression, discarding more information from them. The reason for doing so is that the human eye perceives more detail in still objects than in moving objects. Consequently, in spite of having a worse PSNR because of greater compression, subjectively, the quality of the video sequences with high motion will still be acceptable. To this extent, the video sequences with higher motion, Foreman and Mobile QCIF, obtained after the simulation using a 5–3 (WO–AO) configuration for the 6 × 6 scenario can be seen [38], where, although its quality is rated as “very annoying” according to the PSNR to MOS conversion, the video can be considered as acceptable from a subjective point of view.

### 5.7. Scalability

**Figure 17:**
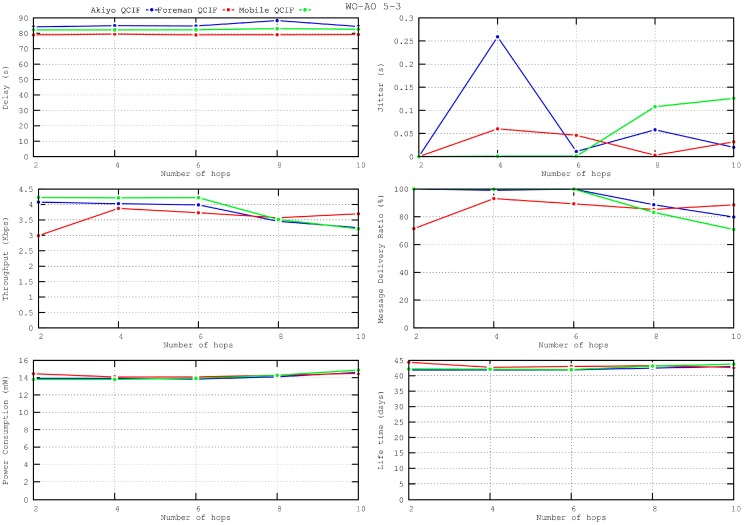
Performance metrics according to the size of the network, WO–AO = 5–3.

The evolution of the obtained performance metrics according to the size of the networks is shown in Figure 17, ranging from 2 hops for a 2 × 2 network to 10 hops for a 6 × 6 network. All metrics remain within acceptable values for any network size, proving the scalability of the IEEE 802.15.5 SES mechanism for video transmission. For the largest network sizes, though, there is a small drop in performance. In these cases, increasing the value of the WI parameter will improve the quality of the received video at the expense of throughput.

## 6. Discussion

The previous section has shown the performance of IEEE 802.15.5 mesh networks transmitting with a packet generation rate of 53 kbps and dispatching one packet per *wakeup interval*, since transmitting more than one packet per *wakeup interval* was observed to lead to congestion and therefore worsen the performance of the network. In particular, the effect of using different WO–AO configurations for several network sizes has been studied.

In spite of considering that just a single node is transmitting video, the previous metrics show that there is some drop of performance because different packets of the same stream may be routed by different paths and compete for transmission. In this regard, the performance drop observed is due to repetitive failures to gain access to the medium while it is busy (CSMA-CA failure), aggravated by the exposed and hidden nodes problems. To cope with these problems, the reservation-based method of the SES mode proposes the use of a relatively short *active duration* period, where all contention for reserving transmission slots takes place, and the *inactive duration*, where nodes have slots where transmission is guaranteed. In data-intensive applications, though, this inactive period can also be affected by the hidden node problem to some degree, as mentioned.

During the active period, nodes have the opportunity to reserve slots for subsequent transmissions in the inactive period. Since the active duration of all neighbor nodes is synchronized, it is likely that several nodes will try to perform simultaneous reservations. In order to avoid collisions, the CSMA-CA scheme allows that, if some reservation message cannot be sent because the channel is busy, several subsequent retransmission attempts (up to a configurable maximum number in the IEEE 802.15.4 standard, *macMaxCSMABackoff*, 4 by default) can be performed. However, according to the simulations, AO values of 1 and 2 result in active durations not long enough to perform all CSMA-CA retries. For example, the maximum delay (in a worst case scenario) due to the CSMA-CA backoff is 36.8 ms (calculated as $\overset{backoff=4}{\underset{backoff=0}{\sum }}(2^{BE}-1)*\text{aUnitBackoffPeriod}\;*\;\text{symbol}\_\text{time}$ where BE=min(macMinBE+backoff, macMaxBE)) using the IEEE 802.15.4 default values at 2.4 GHz, whereas for AO = 1 the active duration is only 10 ms long. This implies that several active durations may be necessary in order to reserve one transmission slot. However, during that time, new packets would also require to be transmitted, increasing the contention for medium access (if the packets are received by neighbor nodes) or the queue size of the node, and therefore the performance of the network would get worse as time goes on.

Given all the factors that affect negatively the transmissions during the *active duration*, it is clear that it is very important to select an AO value that allows transmission with an appropriate QoS. It has been observed for all the previous QoS metrics that for AO values equal to or greater than 3, the performance has been quite satisfactory. Taking into account that the AO parameter is detrimental to power consumption and network lifetime, a value of 3 can be used for the scenarios studied without affecting latency, jitter, throughput and message delivery ratio.

Regarding the selection of the *wakeup interval* length, and consequently the WO parameter, it is important to find a balance between power consumption (which improves with high WO values) and throughput (which improves with low WO values). In addition, it has also been observed that the size of the network may affect the quality of the received video due to the hidden terminal problem, and more particularly secondary collisions. Other negative factors such as contention for medium access, the exposed node problem or primary collisions due to the hidden node problem are avoided by the use of transmission in reserved slots of the inactive period. Thanks to this fact, the drop of performance in the *inactive duration* is not important and it is only noticeable for networks of size 5 × 5 and greater. As the size of the networks grows, the number of packet retransmissions in the mesh network increases, and therefore the probability of secondary collisions. For the scenarios studied, a WO value of 5 has shown to provide good video quality without worsening the throughput. This can be better observed by watching the received videos since, as mentioned in the previous section, values of PSNR values may be misleading for videos with high motion. It has to be noted that, due to the tendency of worsening PSNR and packet loss with network size, for networks larger than the studied ones, higher values of WO should be considered. In this case, increasing the WO by one implies a more than twofold increase of the *inactive duration* and the number of slots (see Table 5). Therefore transmissions in the *inactive duration* are sparser and the probability of collision decreases.

In view of the simulation results, it can be affirmed that it is feasible to transmit video using the IEEE 802.15.5 standard. Using appropriate WO–AO values, it can achieve the necessary reliability for a satisfactory video quality. On the other hand, the main drawback of the standard is that it does not achieve high throughput values, which hinders its applicability to real-time video applications, although this is anyway very challenging for multi-hop networks. In order to improve throughput, some enhancements are necessary that allow more than one packet per *wakeup interval* to be reliably sent. This can be achieved by tackling the diverse problems that have been discussed in this section in order to arrange transmissions so that multimedia flows can use more than one slot per *wakeup interval*, but also by applying enhancements in the directions given in the Related Work section. In this line, the standard has an important feature, the possibility of allowing transmissions to any other node of the network, which facilitates the development of collaborative or multi-sink applications.

## 7. Conclusions

In this paper, the transmission of video sequences using the low rate part of the IEEE 802.15.5 standard has been evaluated by means of a new simulation tool which is available in [33]. This tool implements the SES reservation-based method as energy-saving mechanism, following faithfully the standard specification. Furthermore, the simulation framework integrates the SES method and the EvalVid software, which generates compressed QCIF video sequences with different motion, encoded using H.264/AVC. These sequences are transmitted through mesh networks of different sizes. The main results of the simulations have been presented and discussed, highlighting the most important metrics such as latency, jitter, time between packets, message delivery ratio, power consumption, lifetime and PSNR. The importance of the selection of appropriate values for the *active order* and *wakeup order* parameters has been emphasized, observing that for 3≤AO<WO video transmission is feasible. A 5–3 (WO–AO) configuration has been found to be the best for the scenarios studied, although, for larger networks, higher WO values can be considered. The video sequence obtained after the simulation of 5–3 and 7–2 (WO–AO) configurations for Akiyo, Foreman and Mobile QCIF can be found at [38,39,40,41,42,43].

In addition, the developed tool can be used to evaluate different enhancements over the IEEE 802.15.5 standard. In spite of the advantages of this standard reported in this work, such as fault tolerance or the possibility that any node transmits to any other node, there is still room for improvement for data-intensive applications and, more particularly, WMSN applications. In this regard, it is possible to introduce enhancements to the original standard, with no detriment to the interoperability with other IEEE 802.15.5 devices, which take into account the particularities of WMSN and increase its performance for this type of application.

## Figures and Tables

**Table 1:** Related work overview.

Reference	Main Contributions	Routing	MAC	Energy-Saving
Garcia-Sanchez *et al.* [12]	Throughput enhancement Cross-layer optimization	One-hop	802.15.4 compliant	Duty-cycle
Farooq *et al.* [13]	Architecture for QoS provisioning, congestion control	Hierarchical (clustered)	Not applicable	Not applicable
PT [15]	Congestion control, determine link capacity	Hierarchical (cluster-tree)	OMAC	CSMA-CA improvement
Kandris *et al.* [16]	Selectively drop packets	Asymmetric, Hierarchical (clustered)	Not specified	Energy-aware routing
Zaidi *et al.* [17]	Prioritized multipath forwarding	Multipath (path selection by QoS)	Not specified	Energy-aware routing
Teo *et al.* [19]	Interference-minimized multipath	Multipath (throughput enhancement)	802.11(DCF)	Not specified
MR2 [20]	Incremental, interference-aware multipath	Multipath (throughput enhancement)	802.15.4	Turn off interfering devices
GEAM [21]	Interference-aware multipath	Multipath (geographic greedy forwarding)	802.11	Not specified
Bidai *et al.* [22]	Interference-minimized multipath	Multipath (throughput enhancement) ZigBee based	802.15.4	Not specified

**Table 2:** Comparison of bitrate, latency (Latency, see Section 5.1) and PSNR (Peak Signal-to-Noise Ratio (PSNR), objective video (or signal) quality metric, analyzed in Section 5.6) according to CRF.

CRF	Bitrate (kbps)	Latency (s)	Mean PSNR (dB)
10 *(medium-low compression level)*	247	356.704	50.936
24 *(medium-high compression level)*	53	84.042	41.730

**Table 3:** Sender trace file structure.

Frame	Type	Size (bytes)	Packets	Sender Time
1	I	2281	23	0.081
2	P	288	3	0.158
3	P	76	1	0.159
4	P	238	3	0.199
…	…	…	…	…

**Table 4:** Video1.frag file structure.

Time Interval	Size (bytes)	Type	Priority	Maximum Size ^1^
81,000	2281	1	0	100
+77,000	288	2	0	100
+1000	76	2	0	100
+40,000	238	2	0	100
…	…	…	…	…

^1^ The Maximum Size, in bytes, corresponds to the maximum payload defined by the 802.15.5 standard, obtained as aMaxMACPayloadSize − meshcMaxMeshHeaderLength = 118 − 18 = 100, where aMaxMACPayloadSize = aMaxPHYPacketSize − aMinMPDUOverhead = 127 − 9 = 118.

**Table 5:** Active and inactive durations according to WO–AO.

Wakeup Order	Active Order	Active Duration (ms)	Wakeup Interval (ms)	Inactive Duration (ms)	Slots	AD Assignment	ID Assignment
5	1	10	160	150	15	6.25%	93.75%
5	2	20	160	140	14	12.50%	87.50%
5	3	40	160	120	12	25.00%	75.00%
5	4	80	160	80	8	50.00%	50.00%
6	1	10	320	310	31	3.13%	96.88%
6	2	20	320	300	30	6.25%	93.75%
6	3	40	320	280	28	12.50%	87.50%
6	4	80	320	240	24	25.00%	75.00%
6	5	160	320	160	16	50.00%	50.00%
7	1	10	640	630	63	1.56%	98.44%
7	2	20	640	620	62	3.13%	96.88%
7	3	40	640	600	60	6.25%	93.75%
7	4	80	640	560	56	12.50%	87.50%
7	5	160	640	480	48	25.00%	75.00%
7	6	320	640	320	32	50.00%	50.00%

**Table 6:** Simulation parameters.

Parameter	Value
Channel Type	Channel/WirelessChannel
Radio-propagation model	Propagation/TwoRayGround
Physical layer	Phy/WirelessPhy/802_15_4
Medium Access Layer (MAC)	Mac/802_15_4
Antenna model	Antenna/OmniAntenna
Frequency	2.4 GHz
CS Threshold	2.13643 × 10^−7^
RX Threshold	2.13643 × 10^−7^
Coverage range	30 m
Distance between two consecutive nodes	25 m
Routing protocol	TDLS
Energy-saving mechanism	SES: Reservation-based method
Simulation time limit	1500 s (25 min)
Traffic	Akiyo, Foreman and Mobile QCIF (176 × 144 resolution)
Video generation rate/service rate	53 kbps bitrate/1 packet per *wakeup interval*

**Table 7:** PSNR to MOS mapping [37].

PSNR	MOS	Impairment
>37	5 (Excellent)	Imperceptible
31–37	4 (Good)	Perceptible but not annoying
25–31	3 (Fair)	Slightly annoying
20–25	2 (Poor)	Annoying
<20	1 (Bad)	Very annoying
